# *Lactobacillus* Mucosal Vaccine Vectors: Immune Responses against Bacterial and Viral Antigens

**DOI:** 10.1128/mSphere.00061-18

**Published:** 2018-05-16

**Authors:** Jonathan S. LeCureux, Gregg A. Dean

**Affiliations:** a Department of Natural and Applied Sciences, Evangel University, Springfield, Missouri, USA; b Department of Microbiology, Immunology and Pathology, College of Veterinary Medicine and Biomedical Sciences, Colorado State University, Fort Collins, Colorado, USA; UMKC School of Medicine

**Keywords:** *Lactobacillus*, mucosal immunity, mucosal vaccines

## Abstract

Lactic acid bacteria (LAB) have been utilized since the 1990s for therapeutic heterologous gene expression. The ability of LAB to elicit an immune response against expressed foreign antigens has led to their exploration as potential mucosal vaccine candidates. LAB vaccine vectors offer many attractive advantages: simple, noninvasive administration (usually oral or intranasal), the acceptance and stability of genetic modifications, relatively low cost, and the highest level of safety possible. Experimentation using LAB of the genus *Lactobacillus* has become popular in recent years due to their ability to elicit strong systemic and mucosal immune responses. This article reviews *Lactobacillus* vaccine constructs, including *Lactobacillus* species, antigen expression, model organisms, and *in vivo* immune responses, with a primary focus on viral and bacterial antigens.

## Abstract (precis)

Lactic acid bacteria (LAB) have been utilized since the 1990s for therapeutic heterologous gene expression. The ability of LAB to elicit an immune response against expressed foreign antigens has led to their exploration as potential mucosal vaccine candidates.

## INTRODUCTION

Lactic acid bacteria (LAB), alongside other food-based platforms, have been utilized since the 1990s for therapeutic heterologous gene expression (1). The ability of LAB to elicit an immune response against expressed foreign antigens has led to their use as potential candidates as mucosal vaccine vectors. As vaccine vectors, they offer several attractive advantages: simple, noninvasive administration (usually oral or intranasal), the acceptance and maintenance of genetic modifications, low cost, and high safety levels. LAB tend to elicit minimal immune responses against themselves, instead inducing high levels of systemic and mucosal antibodies against the expressed foreign antigen following uptake via the mucosal immune system (2).

LAB for use as vaccine vectors generally include Streptococcus gordonii, Lactococcus lactis, or multiple *Lactobacillus* species. S. gordonii has generally fallen out of use, with a few exceptions (3). L. lactis and *Lactobacillus* spp. have continued to grow in use, with the number of publications continuing to increase. Several excellent reviews of L. lactis vaccines have been published (4–6), as well as articles describing how to generate these recombinant bacteria (7). Because of the large number of recent articles detailing lactobacilli as vaccine vectors, this review focuses on those publications and on the resulting immune responses generated *in vivo*.

Briefly, this review is divided into sections corresponding to the pathogen/disease of interest (virus, bacterium). Pathogen species or families that have been investigated in multiple studies (i.e., human immunodeficiency virus [HIV], Escherichia coli) are then highlighted, focusing on the immune responses resulting from *Lactobacillus* vaccination. This review covers only research involving *Lactobacillus* strains with heterologous gene expression. Studies conducted with unmodified *Lactobacillus* used either as an adjuvant or for intrinsic antibacterial or antiviral properties are excluded (8, 9). The text of this review focuses on *in vivo* immune responses and on selected *in vitro* studies with a significant immune component, with Table 1 highlighting viral antigens and Table 2 highlighting bacterial antigens.

**TABLE 1:** Primary articles describing studies that utilized *Lactobacillus* to express viral antigens^a^

Pathogen	*Lactobacillus* species	Antigen(s) expressed	Expression	Result(s)	Intended host(s)	Reference
CAV	L. acidophilus	VP1	Surface	Serum Ab, T cell response	Poultry	93
CSFV	L. plantarum	E2	Surface	Serum IgG, mucosal IgA, T cell response	Swine	62
CSFV	L. casei	CTL 290	Secreted	Serum IgG, mucosal IgA, T cell response, challenge	Swine	60
CSFV	L. casei	CTL 290	Unknown	Serum IgG, T cell response, challenge	Swine	61
CSFV	L. casei	CTL 290	Secreted	Serum IgG, mucosal IgA	Swine	94
CyHV-3	L. plantarum	ORF81	Surface	IgM, challenge	Fish	59
FMDV	L. casei, L. plantarum	VP1	Intracellular	Serum Ab, mucosal IgA	Human	95
FMDV	L. acidophilus	VP1	Unknown	Serum IgG, T cell response	Animal	66
GPV	L. plantarum	VP2	Unknown	Mucosal sIgA, TNF-α, IFN-γ, T cell response	Poultry	96
HDV	L. casei, L. plantarum	HDVag	Intracellular	Serum Ab, mucosal IgA	Human	95
HIV	L. jensenii	scFv m9, dAb m36, m36.4	Secreted	Stability	Human	19
HIV	L. acidophilus	MPER	Surface	Serum IgG, mucosal IgA, mucosal IgG	Human	13
HIV	L. plantarum	Gag	Surface	*In vitro* T cell line chemotaxis	Human	14
HIV	L. jensenii	CV-N	Secreted	Safety, toxicity	Human	17
HIV	L. acidophilus	Gag	Surface	Mucosal IgA	Human	12
HIV	L. jensenii	CV-N	Secreted	Challenge	Human	16
HIV	L. fermentum	Gp41	Surface	Stability	Human	97
HIV	L. jensenii	CV-N	Secreted	Safety, toxicity	Human	18
HPV	L. casei	E7	Unknown	T cell response	Human	98
HPV	L. casei	L2	Surface	Serum IgG, mucosal IgG, mucosal IgA, challenge	Human	27
HPV	L. casei	E7	Surface	CTL response, challenge	Human	29
HPV	L. casei	E6	Surface	Serum IgG, mucosal IgA, challenge, cross-neutralization	Human	26
HPV	L. plantarum	E7	Surface	Serum IgG, challenge	Human	30
HPV	L. casei	E7	Surface	Serum IgG, mucosal IgA, challenge	Human	25
HPV	L. casei	E7	Surface	T cell response	Human	28
HPV	Unknown	E7	Surface	Unknown	Human	99
HPV	L. casei	L1, VLP	Intracellular	Serum IgG	Human	23
HPV	L. plantarum	E7	Surface	Stability	Human	100
HPV	L. casei	E7	Unknown	Increased cervical lymphocytes, decreased pathology	Human	31
IBDV	L. casei	VP2	Unknown	Serum IgG, mucosal IgA, challenge survival	Poultry	101
IBV	L. salivarius	EpiC	Surface	Stability, toxicity	Poultry	102
IBV	L. salivarius	EpiC	Secreted	Stability	Poultry	102
Influenza virus	L. casei	NP	Unknown	Stability	Human	103
Influenza virus	L. casei	sM2, HA2	Surface	Serum IgG, mucosal IgA, challenge	Human, animal	37
Influenza virus	L. casei	sM2	Surface	Serum IgG, mucosal IgA, T cell response, challenge	Human	36
Influenza virus	L. delbrueckii	HA	Unknown	Serum IgG, mucosal IgA, challenge	Poultry	35
Influenza virus	L. casei	NP	Unknown	Stability	Human	104
Influenza virus (H1N1)	L. plantarum	M2e	Unknown	Mucosal IgA, T cell response, challenge	Human, swine	105
Influenza virus (H5N1)	L. acidophilus, L. delbrueckii	HA	Unknown	Serum IgG, mucosal IgA	Human, poultry	34
Influenza virus (H9N2)	L. plantarum	HA	Unknown	Serum IgG, mucosal IgA, T cell response, challenge	Poultry	33
Influenza virus (H9N2)	L. plantarum	NP, M1	Unknown	Serum IgG, mucosal sIgA, T cell response, challenge	Poultry	106
Influenza virus (H9N2)	L. plantarum	HA	Unknown	Serum IgG, mucosal IgA, T cell response, challenge	Poultry	32
Influenza virus (H9N2)	L. plantarum	NP, M1	Unknown	Mucosal IgA, T cell response, challenge	Poultry	107
Influenza virus (H5N1)	L. casei	NS1	Surface	Stability	Human Poultry	108
IPNV	L. casei	VP2, VP3	Surface, secreted	Serum IgM, challenge protection	Fish	58
IPNV	L. casei	VP2	Surface, secreted	Serum IgM, challenge	Fish	57
NDV	L. plantarum	HN	Unknown	Serum IgA, mucosal IgA, T cell response, challenge	Poultry	65
Norwalk virus	L. casei	VP60	Intracellular	Stability	Human	109
PEDV	L. casei	COE	Surface	Serum IgG, mucosal IgA, T cell response, > neutralization	Swine	45
PEDV	L. casei	S1, N	Surface, secreted	Serum IgG, mucosal IgA	Swine	44
PEDV	L. casei	N	Surface	Serum IgG, mucosal IgA	Swine	110
PEDV	L. casei	N	Surface	Serum IgG, mucosal IgA	Swine	46
Porcine RV	L. casei	VP4	Surface	Serum IgG, mucosal IgA, neutralization	Swine	53
Porcine RV	L. acidophilus	VP7	Unknown	Mucosal IgA, challenge	Swine	111
Porcine RV	L. casei	VP4	Unknown	Serum IgG, mucosal sIgA, neut. Ab	Swine	112
PPV	L. casei	VP2	Secreted	Serum IgG, mucosal IgA, T cell response, challenge	Swine	60
PPV	L. casei	VP2	Surface, secreted	Serum IgG, mucosal IgA	Swine	64
PPV	L. casei	VP2	Secreted	Serum IgG, mucosal IgA	Swine	94
PPV	L. casei	VP2	Surface	Serum IgG, mucosal IgA	Swine	63
RV	L. rhamnosus	ARP1	Surface	Challenge	Human	54
RV	L. paracasei	ARP1–ARP3	Surface, secreted	Stability	Human	113
RV	L. rhamnosus	IgGb, IgGd	Surface	Challenge	Human	55
SARS-CoV	L. casei	SA, SB	Surface	Serum IgG, mucosal IgA	Human	42
SVCV	L. plantarum	GP	Surface	IgM, challenge	Fish	59
TGEV	L. casei	D	Surface	Serum IgG, mucosal IgA, T cell response, challenge	Swine	47
TGEV	L. casei	MDP	Surface	Serum IgG, mucosal IgA, T cell response, neutralization	Swine	41
TGEV	L. pentosus	6D	Surface, secreted	Serum IgG, mucosal IgA	Swine	40
TGEV	L. casei	S	Secreted	Serum IgG, mucosal IgA	Swine	39

a CAV, chicken anemia virus; CyHV-3, cyprinid herpesvirus 3; FMDV, foot-and-mouth disease virus; GPV, goose parvovirus; HDV, hepatitis D virus; IBDV, infectious bursal disease virus; IBV, infectious bronchitis virus; NDV, Newcastle disease virus; Porcine RV, porcine rotavirus; PPV, porcine parvovirus; SVCV, spring viremia of carp virus; Ab, antibody; neut. Ab, neutralizing antibody; sIgA, secretory immunoglobulin G; scFv, single chain variable fragment.

**TABLE 2:** Primary articles describing studies that utilized *Lactobacillus* to express bacterial antigens^a^

Pathogen	*Lactobacillus* species	Antigen(s) expressed	Expression	Result(s)	Intended host(s)	Reference
Bacillus anthracis	L. gasseri	PA	Unknown	Serum IgG, mucosal IgA, T cell response	Human, animal	71
Bacillus anthracis	L. gasseri	PA	Unknown	Neutr. Ab, T cell response, challenge	Human	70
Bacillus anthracis	L. acidophilus	PA	Surface	Neutr. Ab, mucosal IgA, challenge	Human	69
Bacillus anthracis	L. casei	PA	Surface, intracell., secreted	Serum IgG	Human	68
Bacillus anthracis	L. acidophilus	PA	Surface	Stability	Human	114
Borrelia burgdorferi	L. plantarum	OspA	Surface	Serum IgG, mucosal IgA	Human	83
Borrelia burgdorferi	L. plantarum	OspA	Unknown	Serum IgG, mucosal IgA, challenge	Human	82
Bordetella pertussis	L. casei	FHA	Intracell.	Serum IgG	Human	115
Clostridium botulinum	L. acidophilus	BoNT/A-Hc	Surface	Stability	Human	114
Clostridium perfringens	L. casei	ε-Toxoid	Surface	Serum IgG, serum IgA, intestinal IgA, IFN-γ, challenge	Human, animal	86
Clostridium perfringens	L. casei	α-, β1-, β2-, ε-toxoids	Unknown	Serum IgG, fecal IgA, nasal IgA, IFN-γ/IL-4, T cell response, challenge	Human, animal	116
Clostridium perfringens	L. casei	β-Toxoid	Surface, intracell.	Serum IgG, serum IgA, intestinal IgA, IFN-γ, challenge	Human, animal	117
Clostridium perfringens	L. casei	α-Toxoid	Surface	Serum IgG, mucosal IgA, challenge	Human, animal	118
Chlamydia psittaci	L. fermentum	OmpA	Surface	Stability	Animal	97
Clostridium tetani	L. casei	TTFC	Surface, intracell., secreted	Serum IgG	Human	119
Clostridium tetani	L. plantarum	TTFC	Intracell.	Serum IgG, mucosal IgA	Human	120
Clostridium tetani	L. plantarum	TTFC	Intracell.	Serum IgG	Human	121
Clostridium tetani	L. johnsonii	TTFC	Surface	Serum IgG, mucosal IgA	Human	122
Clostridium tetani	L. plantarum	TTFC	Intracell., secreted, surface	Serum IgG, mucosal IgA	Human	123
Clostridium tetani	L. plantarum	TTFC	Intracell.	Serum IgG, mucosal IgA, T cell response, challenge	Human	124
Clostridium tetani	L. plantarum, L. casei	TTFC	Intracell., surface	Serum IgG, mucosal IgA, T cell response	Human	125
Chlamydia trachomatis	L. plantarum, L. fermentum	VD4	Surface	Stability	Human	126
Chlamydia trachomatis	L. plantarum	Hirep2	Surface	Serum IgG, serum IgA, mucosal IgA, IFN-γ	Human	90
Escherichia coli (EHEC O157:H7)	L. acidophilus	EspA, Tir	Secreted	Serum IgG, mucosal sIgA, IFN-γ, IL-4, IL-10, challenge	Human	127
Escherichia coli (EPEC)	L. casei	β-Intimin	Unknown	Serum IgG, mucosal IgM, challenge	Human	77
Escherichia coli (ETEC)	L. casei	K88	Unknown	Serum IgG, mucosal sIgA, challenge	Human	128
Escherichia coli (ETEC)	L. casei	FaeG	Secreted	Stability	Human	129
Escherichia coli (ETEC)	L. casei	FP	Secreted	Stability	Human	129
Escherichia coli (ETEC)	L. casei	F1	Surface	Serum IgG, mucosal IgA, challenge	Swine, ruminants, human	75
Escherichia coli (ETEC)	L. casei	K88, K99	Surface	Serum IgG, mucosal IgA, T cell response	Swine, ruminants, human	74
Escherichia coli (ETEC)	L. casei	K99	Surface	Serum IgG, mucosal IgA	Swine, ruminants, human	73
Escherichia coli (ETEC)	L. casei	F41	Surface	Serum IgG, mucosal IgA, T cell response	Swine, ruminants, human	72
Escherichia coli (ETEC)	L. reuteri	ST, LT(B)	Secreted	Serum IgG, mucosal IgA, challenge protection	Swine, ruminants, human	76
Escherichia coli (ETEC)	L. acidophilus	K99	Surface	*In vitro* inhibition of pathogen adhesion	Swine, ruminants, human	130
Escherichia coli (ETEC)	L. plantarum	Fimbrial adhesin (FaeG)	Unknown	Serum IgG, intestinal IgA, challenge	Swine, ruminant, human	131
Escherichia coli (UPEC)	L. reuteri	PapG	Surface	Stability	Human	132
Helicobacter pylori	L. acidophilus	Hp0410	Unknown	Serum IgG, mucosal IgA, challenge	Human	85
Helicobacter pylori	L. acidophilus	Hp0410	Surface	Stability	Human	133
Helicobacter pylori	L. plantarum	UreB	Unknown	Serum IgG, serum IgA, challenge	Human	134
Mycobacterium avium (MAP)	L. salivarius	MMP	Surface	Stability	Ruminant	135
Mycobacterium avium (MAP)	L. salivarius	ptD	Intracell.	Stability	Ruminant	136
Mycobacterium tuberculosis	L. plantarum	Ag85B, ESAT-6	Surface	Mucosal IgA, T cell response	Human	137
Salmonella enterica (SE)	L. casei	FliC, SipC	Surface	Serum IgG, T cell response	Human, animal	138
Salmonella enterica (SE)	L. casei	FliC	Surface	Challenge	Human	139
Streptococcus mutans	L. zeae	scFv	Surface, secreted	Challenge	Human	140
Streptococcus pneumoniae	L. casei	PspC	Surface, intracell.	Mucosal IgA, challenge	Human	141
Streptococcus pneumoniae	L. casei	PspA, PspC	Surface	Serum IgG, mucosal IgA	Human	81
Streptococcus pneumoniae	L. casei	PspA	Surface	Serum IgG, challenge	Human	80
Streptococcus pneumoniae	L. casei, L. plantarum, L. helveticus	PspA	Surface	Serum IgG, mucosal IgA, challenge	Human	79
Streptococcus pneumoniae	L. casei	PsaA, PspA′1, PspA′3	Intracell., secreted	Stability	Human	142
Streptococcus pyogenes	L. gasseri	CRR6	Unknown	Serum IgG, mucosal IgA, challenge	Human	143
Streptococcus pyogenes	*L. sake*, L. fermentum	M6	Secreted, surface	Stability	Human	144
V. cholerae	L. casei, L. reuteri	CTB	Intracell., secreted	Serum IgG	Human	145
Vibrio parahaemolyticus	L. rhamnosus	MAM-7	Unknown	MAM-7 expression (reduced *Lactobacillus* ability to inhibit pathogen)	Human	146
Vibrio parahaemolyticus	L. rhamnosus	MAM-7	Unknown	Stability	Human	146
Yersinia pestis	L. plantarum	LcrV	Surface	Serum IgG, mucosal IgA, T cell response	Human	84
Yersinia pseudotuberculosis	L. plantarum	D1-D5, D4-D5	Surface	Stability	Human	147

a FHA, filamentous hemagglutinin adhesin; BoNT, clostridial botulinum neurotoxin; TTFC, tetanus toxin fragment C; FP, fusion protein; MMP, mucous membrane pemphigoid; intracell., intracellular; Neutr. Ab, neutralizing antibody.

## VIRUSES

### Human immunodeficiency virus.

Human immunodeficiency virus type 1 (HIV-1) has been relegated to the status of being a treatable chronic disease, and yet infection rates are unacceptably high (10). An effective HIV vaccine is still elusive via traditional methods, with statistical significance limitations plaguing the only modestly successful clinical trial (11). Utilizing lactobacilli as mucosal vaccine vectors can provide an enhanced immune response at the typical mucosal sites of infection. Several studies have looked at lactobacilli expressing HIV antigens, thus targeting the virus at the most common site of infection, namely, the mucosa. Our laboratory has shown that expressing additional secreted molecules as adjuvants (interleukin 1β [IL-1β], *Salmonella* flagellin C) can significantly improve the mucosal (IgA) and systemic (serum IgG) immune responses against HIV proteins (MPER, Gag) in orally dosed mice (12, 13). Kuczkowska et al. have shown *in vitro* evidence of T cell recruitment using an L. plantarum strain expressing a fusion protein of CCL3/HIV Gag (14). No challenge studies in monkeys or humans have been performed to determine the efficacy of the immune response.

An alternative preventative measure against HIV is the use of prophylactic topical microbicides, which can be effective in high-risk groups (15). By incorporating microbicide expression into lactobacilli, mucosal sites can be colonized and continuously protected, reducing cost and the need for strict adherence. In two separate studies, Lagenaur et al. utilized a vagina-associated L. jensenii strain secreting cyanovirin-N, a promising microbicide with high affinity for HIV envelope glycoproteins. This application was safe in rhesus macaques and afforded protection against simian-human immunodeficiency virus (SHIV) challenge (16–18). That group also used lactobacilli for secretion of broadly neutralizing antibody fragments to protect the vaginal mucosa, though the work was still performed *in vitro* (19). Human trials are under way.

### Human papillomavirus.

The association between human papillomavirus (HPV) and various cancers, particularly cervical cancer, is well known (20). Because of this association, HPV proteins are usually expressed on the surface cervical cancer cells. This allows an immune response that not only targets potentially infectious virus but can also destroy infected, cancerous cells. There are currently two FDA-approved vaccines against the most common strains of HPV (vaccines Gardasil and Cervarix). Both generate protective immune responses via spontaneous virus-like particle (VLP) formation of the HPV L1 capsid protein (21). While these vaccines provide excellent protection and represent potential cancer therapies, the cost can prove prohibitive even in the United States (22). Only one research group has utilized *Lactobacillus* to generate VLPs using the L1 protein, resulting in serum IgG expression following subcutaneous injection in BALB/c mice (23). All other research groups have utilized surface expression of HPV proteins, either minor capsid protein L2 or the early oncoproteins E6 and E7, which are directly responsible for unregulated cellular replication (24). In an extensive set of early experiments, Poo et al. utilized an E7-expressing L. casei strain, observing serum IgG along with intestinal and vaginal IgA in orally immunized C57BL/6 mice. They also observed E7-specific gamma interferon (IFN-γ)-secreting cells in the vagina and spleen, as well as a therapeutic reduction in tumor size and increased animal survival following TC-1 tumor cell challenge (25). A similar study using E6 had similar results (26). Poo et al. later targeted the L2 protein in BALB/c mice, observing serum IgG, mucosal IgG and IgA, and cross-neutralization with related viruses (27). Using L. casei administered to C57BL/6, Adachi et al. observed increased levels of E7-specific T cells in the gut, as well as granzyme-B production. Mucosal lymphocytes were found to be capable of TC-1 cell lysis, a result which was also repeated by another research group (28, 29). Interestingly, oral administration improved the response in comparison to the results seen with subcutaneous or intramuscular administration (28). Another research group utilized L. plantarum expressing E7, with similar antibody and antitumor results, though they checked only for antibodies in the serum and not in the mucosa (30). Because of the observed therapeutic effect seen in several studies, a human trial using cervical cancer (cervical intraepithelial neoplasia grade 3 [CIN3]) patients was conducted and demonstrated the presence of E7-specific lymphocytes in cervical tissues but not in blood, with the majority of patient tumor pathologies being downgraded (31). Taken together, the data show great promise and potential for the development of anti-HPV *Lactobacillus* vaccines to meet an important public health need.

### Influenza virus.

The unpredictability of the availability of future influenza virus strains, as well as supply problems stemming from slow growth methods (egg and cell based), means that anti-influenza *Lactobacillus* vaccines could fill a need, particularly for treatment of infections by highly pathogenic strains such as H5N1. Shi et al. showed that oral administration of an L. plantarum strain expressing H9N2 hemagglutinin (HA) induced fecal IgA, bronchiolar IgA, and serum IgG. B cell levels in secondary lymphoid organs were increased, and CD8^+^ T cell proliferation and IFN-γ secretion were greatly improved relative to the levels seen with a typical influenza vaccine. Most importantly, vaccinated mice survived lethal challenge (32). These results were seen again in assays using dendritic cell-targeting peptide (DC-pep) adjuvant, which showed improved immune responses and challenge survival in chickens (33). Similar antibody and T cell results were observed in targeting H5N1 hemagglutinin (HA_1_) in BALB/c mice (34) and chickens (35). Other influenza virus proteins have also been targeted. Chowdhury et al. granted BALB/c mice protection (via oral or intranasal administration) from multiple lethal challenge strains and showed that inclusion of cholera toxin subunit A1 (CTA1) significantly improved antibody levels and protection (36). A follow-up study showed that antibody levels and IFN-γ secretion and proliferation, as well as protection against lethal challenge, lasted 7 months postvaccination (37).

### Coronavirus.

Until the recent outbreaks of severe acute respiratory syndrome (SARS) (2003) and Middle East respiratory syndrome (MERS) (2014/2015), coronavirus (CoV) morbidity and mortality were generally worse for domesticated animals rather than for humans, particularly within porcine and poultry farms. Coronaviruses usually infect via the gastrointestinal tract in livestock and the respiratory tract in birds and humans, causing devastating economic losses and dangerous morbidities in the young, old, and immunocompromised (38). The first coronavirus addressed using lactobacilli was transmissible gastroenteritis coronavirus (TGEV), which affects swine, particularly piglets. Several spike protein epitopes have been targeted (S, 6D), resulting in induction of serum IgG and mucosal IgA in mice (39, 40). More recently, the muramyl dipeptide (MDP) protein was targeted, utilizing tuftsin as an adjuvant, and the results showed improved antibody and T cell responses in BALB/c mice (41). The only human coronavirus addressed was SARS-CoV, with induction of serum IgG and mucosal IgA against spike proteins (SA, SB) observed in C57BL/6 mice (42). Porcine epidemic diarrhea virus (PEDV) is another coronavirus that primarily affects piglets, resulting in large economic losses (43). In a thorough set of experiments, Liu et al. showed that, by targeting both the spike protein (S1) and nucleocapsid (N) via surface expression (rather than via secretion), levels of anti-S1 and anti-N antibodies were significantly increased, even in atypically studied secretions such as ophthalmic and nasal secretions (44). Interestingly, they observed a synergy against the spike protein, but not against the nucleocapsid, in mice vaccinated against both proteins.

To improve the immune response against TGEV’s core neutralizing epitope (COE), Ge et al. fused the COE with E. coli enterotoxin B (LTB), with results which showed some statistical significance, particularly with respect to splenocyte IFN-γ and IL-4 secretion (45). In perhaps the most directly useful study, Hou et al. observed the increased presence of anti-nucleocapsid antibodies in the milk and colostrum of nursing sows, correlating with increased anti-N serum IgG levels in suckling piglets (46). A recent set of experiments by Jiang et al. delved deeper into the immune response generated by L. casei, highlighted by strong mucosa-dependent protection from infection, stimulation of the IL-17 pathway, and an imbalance between the Th1 and Th2 responses, as indicated by variations in numbers of CD4^+^ T cells containing either intracellular IFN-γ or IL-4 (47). Interestingly, some *Lactobacillus* species have been shown to downregulate IL-17 responses (48), but this simply points to the delicate balance that Th17 cells must strike between pathogen-stimulated inflammation and the potential damage of errant autoimmune inflammation (49). It is clear that homeostasis with respect to inflammation, immunity, lactobacilli, and Th17 cells is a complex subject and is dependent on a number of factors, including host genetics, pathogen, *Lactobacillus* strain, and adjuvants.

### Rotavirus.

Diarrheal disease is the second leading cause of death in children under the age of 5 worldwide, with rotavirus responsible for 40% of hospitalizations due to diarrheal illness (50). It is estimated that rotavirus killed approximately 215,000 children in 2013. The World Health Organization recommends inclusion of a rotavirus vaccine in all global vaccination protocols, and there are currently two modified live vaccines licensed worldwide (51). The global implementation is ongoing, but in countries where data are available, vaccination has resulted in a 33% reduction in hospitalization due to rotavirus morbidities. Unfortunately, both vaccines have limited (50% to 60%) efficacy in developing countries and are associated with a low-level risk of intussusception (52). A recombinant *Lactobacillus*-based vaccine could address the need for a subunit rotavirus vaccine that provides the benefits of a probiotic and the appropriate safety profile for use in neonates and infants. Two main avenues of lactobacillus-based rotavirus protection have been attempted in mice. The first avenue used typical oral vaccination with L. casei, inducing mucosal IgA and neutralizing serum IgG against porcine Rotavirus major protective antigen (PA) VP4 in mice (53). The second used antibody fragments to confer protection. Álvarez et al. expressed a protective anti-rotavirus llama antibody fragment on the surface of L. rhamnosus, protecting against diarrhea in a mouse pup model (54). Another group adapted the use of anti-rotavirus hyperimmune bovine colostrum (HBC) in the same model system, expressing an anti-HBC protein from *Streptococcus*, which binds HBC antibodies, thus conferring protection when orally dosed (55).

### Fish-related viruses.

Aquaculture is an important food supply paradigm, and with it comes the typical pathogen problems that large-scale animal farms encounter. Vaccination against fish pathogens can be performed by intraperitoneal administration (which can be cost-prohibitive), by immersion, or orally via feed, with the latter two options suffering from a lack of vaccine persistence in water and from the particularly strong mucosal tolerance observed in fish. For a comprehensive summary of vaccination attempts in fish, see the excellent review by Embregts and Forlenza (56). Lactobacillus vaccine vectors can provide an effective and easily administered system for pisciculture. The first set of studies targeted infectious pancreatic necrosis virus (IPNV), a birnavirus that afflicts rainbow trout. Direct oral administration with L. casei expressing portions of viral capsid generated significant serum IgM and afforded challenge protection in two studies by the same group (57, 58). Two viruses that primarily affect carp, *Cyprinid herpesvirus 3* (Koi herpesvirus [KHV]) and Rhabdovirus carpio (spring viremia of carp virus [SVCV]), have also been studied. The two antigens (KHV ORF81 and SVCV glycoprotein) were expressed together in L. plantarum and dosed orally in carp and koi. The resulting serum IgM and challenge survival data were promising, particularly for a vaccine that offers dual protection (59). Further *Lactobacillus* studies must be conducted, looking in particular at cellular mucosal immunity in fish, as well as at the potential for multiple pathogens to be addressed with a single modified *Lactobacillus* vaccine.

### Other viruses.

In addition to the categories already addressed, a large and diverse number of viruses have been targeted using *Lactobacillus* vector systems. A few are highlighted here, with the rest detailed in Table 1. Classical swine fever virus (CSFV), a flavivirus affecting pigs, has been tested in rabbits, mice, and pigs, with all tests resulting in production of serum and mucosal antibodies (60, 61). Importantly, addition of thymosin α-1, a T cell-stimulating peptide, was able to increase levels of IgG, IgA, IFN-γ, IL-2, and tumor necrosis factor alpha (TNF-α) in pigs (62). *Porcine parvovirus* has been studied in BALB/c mice and pigs, with excellent IgG and IgA responses, as well as challenge protection and virus neutralization (60, 63, 64). A recent study observed strong protective immune responses in chickens against *Newcastle disease virus*, a paramyxovirus primarily afflicting poultry, which were improved by the addition of DC-pep, which not only boosted mucosal and serum antibody levels but also increased levels of T helper cells in the spleen and peripheral blood versus the results seen with bacteria without DC-pep (65). Foot-and-mouth disease virus, a *Picornavirus* afflicting cloven-hooved animals, was investigated in a comprehensive dosing study that assessed anticapsid immune responses resulting from administration of recombinant L. acidophilus via the intramuscular, intraperitoneal, intranasal, or oral route. Of note, this vaccine strategy utilized the bacteria as a delivery vehicle for a capsid-expressing DNA vaccine plasmid, in contrast to utilization of expression of heterologous proteins by the bacteria. The resulting antibody responses were thus much higher via intramuscular and intraperitoneal administration than via mucosal delivery (66). As the ease of use and awareness of *Lactobacillus* expression systems and their abilities to induce excellent mucosal and systemic immune responses increase, the number and variety of pathogens addressed will likely increase in the future.

## BACTERIA

### Bacillus anthracis.

Though infections are relatively rare, the prevalence of natural Bacillus anthracis in soil and its potential as a bioterrorist agent gives antianthrax vaccines some priority. Protective antigen (PA), the only antigen used in *Lactobacillus* vaccinations, is well studied and has been tested in other vaccine systems with various degrees of success (67). One of the earliest proof-of-concept *Lactobacillus* experiments involved dosing BALB/c mice with L. casei either orally or intranasally. That early study showed that the antibody responses against heterologous protein exceeded the antibody responses against the bacteria itself (68). Ten years later, Mohamadzadeh et al. combined an L. acidophilus or L. gasseri strain with DC-pep, resulting in neutralizing antibodies and challenge survival in A/J mice (69, 70). That same group later observed colonic DC activation, Th17 and regulatory T cell (Treg) upregulation, and upregulation of pattern recognition receptor genes with a single vaccine dose (71).

### Escherichia coli.

Enteric Escherichia coli bacteria are a major cause of diarrheal morbidity and mortality, particularly for children in developing countries. The most common antigens targeted for E. coli vaccination are fimbrial proteins, which are bacterial adhesins that aid in host cell binding. Most experiments mentioned here, except one, have targeted enterotoxigenic E. coli (ETEC). A prolific group from China utilized several fimbrial protein antigens (F41, K99, K88) over several years and in several models (BALB/c, C57BL/6, BALB/c pups), all using L. casei. Among their many findings, an increase in levels of several subclasses of serum IgG (IgG1, IgG2a, IgG2b) followed oral dosing, along with increased IL-4 levels and a lesser increase of IFN-γ levels measured by CD4^+^ T cell enzyme-linked immunosorbent spot (ELISPOT) assays. Intestinal and bronchiolar IgA levels were increased, and challenge with standard ETEC resulted in protection of >80% of mice challenged with a lethal dose (72). The studies were repeated using intranasal dosing, which resulted in decreased intestinal IgA levels but increased bronchiolar IgA levels compared to oral delivery (73). Dosing in C57BL/6 mice induced similar IgG and IgA responses, as well as T cell proliferation and challenge protection (74). Challenge protection was conferred to mouse pups born to orally or intranasally immunized dams (75). Wu and Chung targeted two enterotoxins (ST and LT-B), rather than fimbrial proteins, with a secreted green fluorescent protein (GFP)/enterotoxin fusion protein. Similar increases in IgG and IgA levels were observed as well as challenge protection in a patent mouse gut assay (76). Ferreira et al. were the only group to target enteropathogenic E. coli (EPEC) and attempted the only sublingual dosing regimen. Experiments using L. casei expressing a portion of bacterial β-intimin (a cell surface protein that aids in attachment to the host cell) resulted in serum IgG and fecal IgA responses, though, interestingly, oral dosing did not generate an IgG response. Splenocytes also secreted elevated levels of IL-6 and IFN-γ, though only the results from the sublingual vaccination were reported (77). While Ferreira et al. performed their studies in C57BL/6 mice, they used C3H/HePas mice as their challenge model, due to that strain’s susceptibility to Citrobacter rodentium, a commonly used strain that shares some pathology with EPEC (78). Ferreira et al. observed an increase in survival time, though animals eventually succumbed to disease.

### Streptococcus pneumoniae.

Most *Lactobacillus* experiments involving Streptococcus pneumoniae have been performed by the Oliveira laboratory and have focused on pneumococcal surface proteins (either PspA or PspC), with immunity studies conducted in C57BL/6 mice. Early work noted significant increases in bronchiolar IgA but not IgG levels following intranasal administration, with some variations due to bacterial strain differences (79). Strategies to increase antigen expression resulted in increased IgG levels (IgA levels were not measured), with enhancement of multiple IgG subsets (IgG1, IgG2a, IgG2b, IgG3). Challenge survival was improved compared to that seen with controls inoculated with saline solution alone, but no differences from the results seen with animals immunized with bacteria expressing the empty vector plasmid were observed (80). Further experiments identified a propensity for responses involving IgG1 versus IgG2a, which, along with increased IFN-γ levels and low levels of IL-5, indicated Th1 polarization. The levels of IL-17 secretion and neutrophil recruitment in the lungs varied by route of administration, adding to the idea of the importance of the manner in which vaccines are administered and not just of their expression of antigens (81). A final set of experiments failed to induce significant levels of IgA prior to challenge, but the researchers noted that challenge with S. pneumoniae did induce a significant IgA response, which correlated with reduced bacterial loads.

### Other bacteria.

Very few of the large number of pathogenic bacterial species have been targeted with lactobacilli, and such studies have been reported in only a few research publications. A few are highlighted here, with the rest addressed in Table 2. Borrelia burgdorferi, the causative agent of Lyme disease, was targeted with an L. plantarum system, resulting in 100% protection following a B. burgdorferi-infected tick challenge (82). Those authors also identified what has become an interesting theme with lactobacillus vaccinations, i.e., that of dual Th1 and Th2 induction. *In vitro* work with human cells resulted in Th1 and Th2 cytokine responses, and oral administration in C3H-HeJ mice resulted in induction of both IgG1 (Th2) and IgG2a (Th1) (83). The same authors also targeted Yersinia pestis with L. plantarum, observing once again both inflammatory (TNF-α, IL-12, IFN-γ, and IL-6) and anti-inflammatory (IL-10) cytokines, indicating stimulation of both Th1 and Th2 responses (84). Importantly, however, as with the previous experiment, those were human *ex vivo* cytokine studies whose results were not confirmed *in vivo*. A vaccine targeting Helicobacter pylori, a common cause of stomach ulcers, would be extremely beneficial. By targeting H. pylori adhesin Hp0410 with an L. acidophilus strain, Hongying et al. generated anti-adhesion serum IgG and intestinal IgA that reduced bacterial load and gastric inflammation following challenge (85). Antibodies against the ε-toxoid of Clostridium perfringens were identified in BALB/c mice following oral L. casei administration, and though the statistical significance of the antibody levels was unclear, the animals survived challenge (86).

## CONCLUSIONS

In order to combat most pathogens at their main point of entry, next-generation vaccines must establish protective mucosal immunity (87). Lactic acid bacteria, particularly species of genus *Lactobacillus*, have shown great promise as mucosal vectors that are capable of driving both systemic and mucosal responses, especially in combination with adjuvants. The number of studies involving lactobacilli has steadily increased over the last 20 years, and as data accumulate, key concepts regarding the immune responses that these vectors elicit have emerged. Interestingly, coinduction of Th1 and Th2 cytokines points to the complexity of T cell subsets in the mucosa. A growing number of studies have suggested that T cell effector plasticity in the mucosa, especially in the gut, is the norm and that the gut must strike a balance between tolerance and inflammation (88). This appears to be one major factor arising from these *Lactobacillus* studies, since evidence of Th17 inflammation, as well as of Treg-based tolerance, points to a complex T cell response. In terms of mucosal vaccination, this reiterates the importance of maintaining a balanced and well-characterized approach to immunogenicity. More work must be done to identify the contributing immune pathways within the mucosa, especially the routes of bacterial uptake into immune inductive sites (M cells, DCs).

There are several major takeaways as development of LAB vaccine platforms continues. While the safety of LAB is an important strength, enhancing protective immunogenicity is a key challenge. Several studies have explored strategies to express adjuvants such as cytokines, pathogen-associated molecular patterns, toxins, and targeting molecules for M cells and DCs. A mechanistic understanding of each of these strategies is necessary to design the right combination of immunogens and adjuvants that will result in protection. The route of administration, while typically oral for LAB, can have an effect on the type of response elicited due to differences in mucosal inductive sites. The intrinsic differences between strains of lactobacilli, as well as the location of antigen expression (surface display, intracellular, secreted), can alter the resulting immune response, and the strains must therefore be properly selected for specific antigens (89). Boosting is also clearly a component of successful vaccination, and there is evidence that heterologous prime-boost strategies may improve, or at least alter, the resulting immune response (90). As always, the model system must be taken into consideration, especially in light of new evidence for mucosal immune differences between the two most common mouse models (BALB/c and C57BL/6) (91). On the basis of their safety and efficacy, as well as their overall cost, *Lactobacillus* vaccine vectors hold great promise as mucosal vaccines. It is anticipated that the use of clustered regularly interspaced short palindromic repeat (CRISPR)/Cas9 analysis will allow a more sophisticated approach to engineering vaccine candidates (92). Ultimately, it is critical for one of these candidates to successfully navigate the regulatory gauntlet and demonstrate efficacy in a target population.
